# Functional Genomics for Plant Breeding

**DOI:** 10.3390/ijms222111854

**Published:** 2021-11-01

**Authors:** Fatemeh Maghuly, Beata Myśków, Bradley J. Till

**Affiliations:** 1 Plant Functional Genomics, Institute of Molecular Biotechnology, Department of Biotechnology, BOKU-VIBT, University of Natural Resources and Life Sciences, 1190 Vienna, Austria; 2 Department of Plant Genetics, Breeding and Biotechnology, West-Pomeranian University of Technology, Szczecin, Ul. Słowackiego 17, 71-434 Szczecin, Poland; Beata.Myskow@zut.edu.pl; 3 Veterinary Genetics Laboratory, University of California, Davis, CA 95616, USA; till.brad@gmail.com

To face the rapidly growing world human population, an increase in agricultural productivity and production is necessary to overcome the enhanced food demand. This can be achieved either by increasing the cultivated area or by deploying improved crop plants and yield using next-generation plant breeding. The application of advanced technologies in plants has accelerated the generation of multi-omic data at various levels, such as the genome, proteome, transcriptome, epigenome, and metabolome levels. To take full advantage of this, integrative approaches using mathematical or relational models are needed to sequentially or parallelly combine the available multi-omics data to understand molecular interactions and physiological mechanisms at an organism-wide level [1,2]. In other words, a holistic, integrative approach is often selected to improve interpretation accuracy when addressing the biological question of interest [3]. This offers plant biologists a deeper understanding of plant function and the mechanisms underlying important traits. Omics data have also provided opportunities to explore and link genotypes to phenotypes and, consequently, to narrow the gap between them. Examples can be found in the current Special Issue, “Functional Genomics for Plant Breeding”, which contains seven original articles and three reviews. 

Recent improvements in high-throughput genotyping methods have fueled the development of analytical methods for QTL identification. Macko-Podgórni et al. [4] provide evidence for the likely involvement of DcDCAF1 and DcBTAF1 genes associated with carrot root shape through genome-wide association studies (GWAS). Root morphology is one of the major attributes defining cultivar types, affecting consumers’ choices and food industry needs.

Advances in genomics also illustrate the high complexity of quantitative host–pathogen interactions. Thus, in the current issue, Miedaner et al. [5] review the growing knowledge of the six most important pathosystems, allowing for the development of novel breeding strategies, where population mapping, genomic selection (GS), and integration of genomic data play an important role in accelerating the resistance breeding process in many pathosystems.

Podwyszyńska et al. [6] have estimated the phenotype and genotype changes in a newly obtained tetraploid apple cultivar compared to a diploid cultivar, with a particular interest in increased resistance to apple scab, a severe fungal disease in apple. The tetraploid cultivar showed higher methylation levels and higher expression levels of genes related to different resistance responses.

Furthermore, Kibe et al. [7] have conducted association mapping in conjunction with linkage mapping, joint linkage association mapping, and genomic prediction, to analyze the genetic architecture of common rust (CR) resistance in maize. Based on genotyping-by-sequencing and single-nucleotide polymorphisms markers through GWAS analyses using an association-mapping panel and five F3 biparental populations, they could identify significant marker–trait associations, individually explaining 6–10% of the total phenotypic variances. The obtained data can be used to develop functional molecular markers for marker-assisted selection and implement genomic prediction to improve CR resistance in tropical maize.

Considering that extrinsic and intrinsic sources of stress can damage DNA, plants have developed highly conserved DNA damage–response pathways to protect them from this harmful effect. Jaskowiak et al. [8] analyzed the function of the HvATR gene, a DNA damage-signaling kinase, in response to chemical clastogen-maleic acid hydrazide, using WT and the Al-tolerant barley TILLING mutant hvatr.g. 

Góralska et al. [9] successfully identified the location of the rye gene associated with epicuticular wax formation (glaucousness) using genetic mapping in combination with machine learning (ML). The DNA sequence of DArT-Silico 3585843, closely linked to wax locus segregation, was implicated as one of the candidates controlling the phenotypic trait. This is one example of how genotype–phenotype relationship prediction with GS and ML is becoming a powerful approach in plant genetic and breeding.

Taken together, it is not only the sequence of plant DNA that matters: how do some genes get activated, and why are others silenced? How can genomics facilitate the study of complex traits in plant breeding? In this regard, Salgotra and Stewart [10] review the latest methods used in functional markers and their utilization in precision breeding, quality traits, stress-resistance breeding, marker-assisted selection, and the development of elite crop cultivars. 

Furthermore, RNA sequencing has emerged as a powerful tool for analyzing transcriptomes to identify genes that show differential expression between unstressed and various stress conditions. However, the transcripts obtained from RNA-Seq require reference genome or transcriptome sequences for read-mapping and annotation. In this context, Schaarschmidt et al. [11] used Pacific Bioscience Single-Molecule Real-Time long-read sequencing technology isoform sequencing in rice to identify candidate genes for stress tolerance breeding not present in the reference transcriptomes/genomes. 

The advances in next-generation genome sequencing and the rapid development of cost-effective and easy-to-use genome engineering editing methods have facilitated researchers’ use of these tools for the functional characterization of many genes that are useful for crop improvement. In this regard, genome-editing tools such as CRISPR/Cas9, which can be exploited to explore the genetic resources for improving the various economic traits of crop plants in terms of stress tolerance and nutritional quality, are reviewed by Salava et al. [12]. This review presents various examples of gene editing for simultaneously conferring both biotic and abiotic stress resistance in tomatoes. The prospects and challenges of genome editing, and its social and political acceptance in tomatoes, are also discussed.

Finally, Kloc et al. [13] show that RNAi-mediated silencing of the Glycogen synthase kinase-encoding genes, key players in the brassinosteroid signaling pathway, provide the expected phenotypes and describe their biological functions. Furthermore, the data confirm that modifying the function or expression of these genes is a feasible strategy to generate barley lines with improved agricultural traits under biotic stress conditions. In other words, gene-silencing techniques contributed to an understanding of how genomic variation can be created to produce nutritional benefits in the presence of future environmental changes.

Due to the current demand for nutritional security, the obtained data can be used as a resource to address the need to generate resilience to climate change in crop plants by targeting several traits of interest [1,11]. Therefore, we expect that multi-omic technology-driven functional genomics and computational approaches will play an essential role in the future of plant breeding.

We wish to thank all contributors to this Special Issue and hope that it will raise interest in and understanding of how genomics and related technologies can drive the next generation of plant breeding. Nevertheless, the current Special Issue can only cover a small part of our scope, and since the subject is of the utmost importance, we are proud to announce the opening of the second part of this Special Issue, “Functional Genomics for Plant Breeding 2.0” and invite our readers to follow and contribute to it.
